# Stress-Induced, p53-Mediated Tumor Growth Inhibition of Melanoma by Modulated Electrohyperthermia in Mouse Models without Major Immunogenic Effects

**DOI:** 10.3390/ijms20164019

**Published:** 2019-08-17

**Authors:** Balázs Besztercei, Tamás Vancsik, Anett Benedek, Enikő Major, Mbuotidem J. Thomas, Csaba A. Schvarcz, Tibor Krenács, Zoltán Benyó, Andrea Balogh

**Affiliations:** 1 Institute of Clinical Experimental Research, Semmelweis University, 1097 Budapest, Hungary; 2 1st Department of Pathology and Experimental Cancer Research, Semmelweis University, 1097 Budapest, Hungary

**Keywords:** electrohyperthermia, melanoma, stress response, tumor growth arrest, hsp70, MHC-I

## Abstract

Modulated electrohyperthermia (mEHT), an innovative complementary technique of radio-, chemo-, and targeted oncotherapy modalities, can induce tumor apoptosis and contribute to a secondary immune-mediated cancer death. Here, we tested the efficiency of high-fever range (~42 °C) mEHT on B16F10 melanoma both in cell culture and allograft models. In vivo, mEHT treatment resulted in significant tumor size reduction when repeated three times, and induced major stress response as indicated by upregulated cytoplasmic and cell membrane hsp70 levels. Despite the increased PUMA and apoptosis-inducing factor 1, and moderate rise in activated-caspase-3, apoptosis was not significant. However, phospho-H2AX indicated DNA double-strand breaks, which upregulated p53 protein and its downstream cyclin-dependent kinase inhibitors p21^waf1^ and p27^kip^. Combined in vitro treatment with mEHT and the p53 activator nutlin-3a additively reduced cell viability compared to monotherapies. Though mEHT promoted the release of damage-associated molecular pattern (DAMP) damage signaling molecules hsp70, HMGB1 and ATP to potentiate the tumor immunogenicity of melanoma allografts, it reduced MHC-I and melan-A levels in tumor cells. This might explain why the number of cytotoxic T cells was moderately reduced, while the amount of natural killer (NK) cells was mainly unchanged and only macrophages increased significantly. Our results suggest that mEHT-treatment-related tumor growth control was primarily mediated by cell-stress-induced p53, which upregulated cyclin-dependent kinase inhibitors. The downregulated tumor antigen-presenting machinery may explain the reduced cytotoxic T-cell response despite increased DAMP signaling. Decreased tumor antigen and MHC-I levels suggest that natural killer (NK) cells and macrophages were the major contributors to tumor eradication.

## 1. Introduction

Melanomas, derived from the malignant transformation of melanocytes, show increasing incidence in white-skinned populations, primarily due to enhanced exposure to irradiation of the UV-B and UV-A spectra [1]. Based on Surveillance, Epidemiology and End Result Program (NIH, USA) data, the general five-year survival rate of melanomas is 90.5%. However, it is reduced sharply in cases showing regional (60.3%) or distant (16.2%) spreading. Recent understanding of the molecular background of melanoma development and progression has resulted in promising novel therapies targeting mutant B-Raf enzyme, or the CTLA-4- or PD-1-immune checkpoints in advanced, unresectable cases [1]. Nevertheless, there is still a great need for improving the efficacy of even the most advanced therapy regimens, for which the non-invasive modulated electrohyperthermia (mEHT) could be an available option [2].

Major genetic drivers of melanoma oncogenesis are the gain-of-function mutations of B-Raf (BRAF), neurofibromin 1 (NF1), and NRAS, which contribute to the activation of the ERK/MAPK pathway, resulting in the uncontrolled growth, proliferation, and enhanced survival of tumor cells [1]. Though melanomas express tumor antigens such as melan-A (also known as MART-1, melanoma antigen recognized by T cells) [1], which can adequately signal an adaptive anti-tumor immune response, this can be effectively suppressed by the tumor cells [3]. The best known negative immunomodulators, the PD-L1 and CTLA-4 checkpoint proteins produced by tumor cells, may induce programmed cell death of the effector CD8+ cytotoxic T-cells [4,5]. This knowledge led to the development of the current first-line therapies of inoperable, advanced stage melanomas targeting mediators of the ERK/MAPK pathway: RAF, MEK kinases, or the CTLA-4, PD-1/PDL-1 immune checkpoints using humanized monoclonal antibodies [1]. 

Loco-regional mEHT has been used to improve the efficacy of chemo-, radio-, and, recently, immunotherapy [2,6,7,8]. The capacitive coupling of mEHT generates an electromagnetic field through the tumor-bearing region of the body, which is selectively accumulated in the tumor compared to the adjacent normal tissues [9]. This is driven by the metabolic shift of malignant tissues, resulting in elevated glucose uptake, glycolysis (Warburg effect), lactate, salt, and metal ion concentration [9,10,11,12], leading to increased dielectric permittivity and complex conductivity in the tumor [13,14,15,16]. The selective absorption of the electric field can directly affect dielectric molecules in tumors (mainly concentrated in lipid rafts) and generate heat, which is controlled by the instrument to maintain a temperature of 42 °C [17,18].

The great advantage of mEHT is that it has its own tumor-damaging effects, mediated by irreversible heat and cell stress, and is also highly tolerable for patients, with almost no side-effects [2,19]. In experimental tumor models, we and other groups confirmed that mEHT treatment alone could provoke caspase-independent AIF-mediated and caspase-dependent apoptosis, in association with mutant or wild-type TP53 gene, respectively, in colorectal adenocarcinoma models [20,21,22]. Significant apoptosis and reduced tumor proliferation by this treatment were also reported in glioma cultures [23]. Furthermore, modulated EHT treatment could improve the efficacy of dendritic cell immunotherapy, inducing abscopal effect [24,25]. This effect is likely to be related to the upregulation and release of damage-associated molecular pattern (DAMP) proteins, which were accompanied by progressive immune-mediated secondary tumor-damage (immunogenic cell death) [26]. DAMP signals involve chaperone molecules such as the endoplasmic reticulum (ER)-linked calcium-binding calreticulin and the heat-shock-associated hsp70, as well as the non-histone nuclear protein HMGB1 (high-mobility group box-1) [27]. These were relocalized to the tumor cell cytoplasm, then into cell membranes for final discharge to interact with the immune system [21,26]. As a consequence, progressive tumor infiltration by S100+ antigen-presenting cells, CD3+ T-cells [21], as well as F4/80+ macrophages and eosinophil granulocytes have been observed [24]. The immune-promoting effect of mEHT was also suggested by the negligible number of FoxP3+ regulatory T-cells both in colorectal cancer [21] and in melanoma models [25].

For further understanding and utilization of mEHT treatment, in this work, we investigated its effect and the underlying mechanisms on tumor cell-stress, apoptosis, viability, proliferation, and anti-tumor immunity in a B16F10 melanoma allograft model.

## 2. Results

### 2.1. mEHT Suppressed Melanoma Tumor Growth 

**Figure 1:**
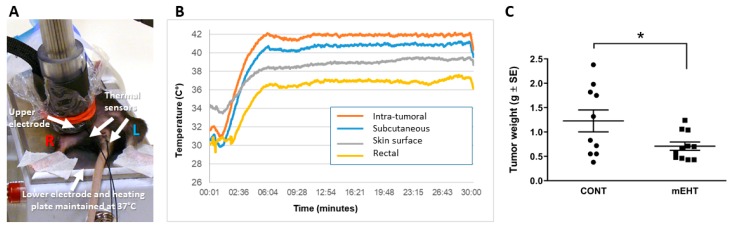
Modulated electrohyperthermia (mEHT) induced reduction of the tumor size. (**A**) Experimental setup of mEHT treatment using LabEHY200. During treatment, mice were laid between plane-parallel asymmetric electric condensers of the circuit which consisted of the lower, 72-cm^2^ aluminum electrode used as a heating plate (set to 37 °C) and the upper telescopic DIA16 conductive textile electrode, which was placed on the tumor. (**B**) Temperature monitoring during mEHT treatment with optical thermo-sensors inserted into the tumor, subcutaneously, rectally, and on the skin surface directly below the upper electrode. (**C**) Tumor weights of the tumors harvested 48 h after the third mEHT treatment, *n* = 10 for control (CONT) and *n* = 11 for mEHT-treated tumors (mEHT). * *p* ≤ 0.03, calculated using unpaired two-tailed *t-*test.

Before performing experiments according to the treatment protocol with the newly developed LabEHY200 device (Figure 1A), we set up conditions providing a standard 42 °C intratumoral temperature in a series of animals. The temperature was monitored using optical thermo-sensors inside tumors, rectally, subcutaneously, and directly below the upper electrode to assess the thermal effect in and around treated tumors (Figure 1B). The power adjustment profile allowed accurate maintenance of temperature around 42 °C in the center of the treated grafts. The subcutaneous temperature was 1 °C lower than the tumor center, while the skin surface was 39 °C, indicating a thermal gradient, with the highest value in the tumor core. Duration of the treatment was 30 min, in accordance with the clinical setting. This pilot adjustment allowed us to avoid invasive temperature monitoring in the following experiments. Using this protocol, relying solely on the skin surface and rectal temperature measurements, we demonstrated that mEHT treatment resulted in significant tumor size reduction when applied three times according to the protocol shown above (Figure 1C). 

### 2.2. mEHT Induced the Expression of Different Forms of hsp70

**Figure 2:**
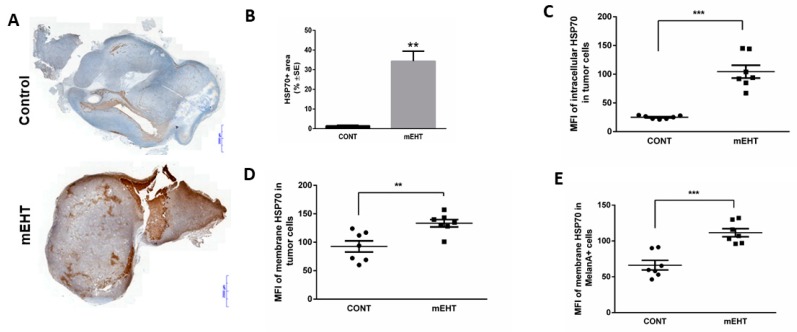
mEHT induced the overexpression of hsp70 protein. (**A,B**) Representative image of tumor sections with hsp70 immunostaining and the corresponding quantitative analysis of the percentage of hsp70+ area detected 24 h after mEHT *n* = 5 for control and *n* = 6 for mEHT, ** *p* ≤ 0.004. Scale bar shows 2000 microns. Flow cytometry analysis 48 h after treatment of (**C**) intracellular, (**D**) membrane-bound, and (**E**) melanocyte-specific membrane-bound hsp70. *n* = 7, ** *p* ≤ 0.004, *** *p* ≤ 0.0008, Mann–Whitney test. MFI: mean fluorescence intensity.

Repeated mEHT treatments at the high-fever range induced a significant stress response in melanoma cells, which we tested by measuring the levels and intracellular distributions of hsp70 protein (Figure 2). Immunohistochemistry showed that 24 h after the third treatment mEHT induced a marked elevation of hsp70 expression (*p* = 0.0043) compared to the control tumors (Figure 2A,B). Furthermore, we measured both the intracellular and the cell-membrane-bound hsp70 levels using selective antibodies and staining procedures to differentiate between them (Figure 2C–E). A ~4-fold increase (*p* < 0.0001) of mean fluorescence intensity (MFI) was detected in the intracellular hsp70 levels of the treated tumor cells (Figure 2C), and a ~1.5 fold increase was measured in the membrane bound form (*p* ≤ 0.004)( (Figure 2D). While the intracellular form of hsp70 may render tumor cells resistant to cytotoxic agents, the membrane-bound protein can support the immunogenicity of the melanocytes. Hsp70 levels were also tested in melan-A-positive cell fractions in order to specify and differentiate melanoma cells from other cellular components of the tumor. The membrane-localized hsp70 protein was elevated both in the whole tumor cell population (*p* < 0.004) and in the pure melanoma cell fractions, and this was more prominent in the latter (*p* < 0.0008) (Figure 2D,E).

### 2.3. mEHT Induced p53 Accumulation and Activation In Vitro and In Vivo

**Figure 3:**
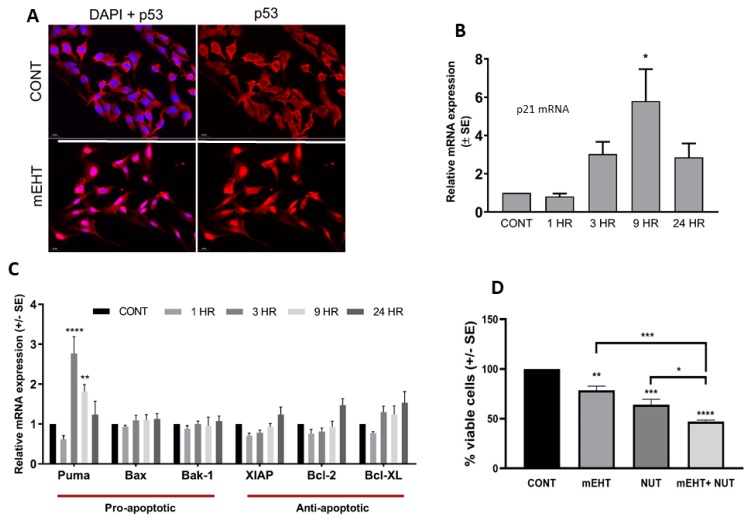
The molecular mechanisms activated by mEHT in B16F10 melanoma cells in vitro. (**A**) mEHT at 42 °C induced p53 activation and nuclear translocation in B16F10 melanoma cells, assayed with ICC 24 h after the treatment (magnification 40×). (**B**) p21 gene expression was measured by qPCR 1, 3, 9, and 24 h after mEHT treatment. (**C**) Time-course of pro- and anti-apoptotic gene expression 1, 3, 9, and 24 h after mEHT treatment measured by qPCR. (**D**) Cell viability determined by MTT assay 24 h post mEHT treatment, using 10 µM nutlin-3a (NUT) alone (mEHT) or combined with mEHT. Cells kept at 37 °C were used as control (CONT). Data are the average of at least three experiments. * *p* < 0.05, ** *p* ≤ 0.001, *** *p* ≤ 0.0001, **** *p* ≤ 0.00001 calculated with one-way ANOVA.

Previous studies have shown that mEHT causes cell death via p53-induced apoptotic response [22]. The B16F10 melanoma cell line maintains functional wild-type p53. Thus, we tested if mEHT acts on p53 in tumor cell cultures using immunocytochemistry to detect the p53 status. Indeed, mEHT treatment caused the upregulation and nuclear translocation of p53, assayed one day after the treatment (Figure 3A). Next, in a time-course experiment, we measured the induction of the canonical p53 target gene CDKN1A (encoding for p21^waf1^ protein) responsible for cell cycle arrest and the expression of pro- and anti-apoptotic genes using qPCR. mEHT resulted in a rapid increase of p21^waf1^ mRNA level, peaking at nine hours after treatment (*p* < 0.008), which bounced back 24 h post-treatment (Figure 3B). In line with p53 activation, the apoptosis-inducer gene PUMA (p53 upregulated modulator of apoptosis) also showed threefold upregulation already 3 h after treatment (*p* < 0.0001), remained elevated at 9 h (*p* < 0.002), then returned to near control levels at 24 h (Figure 3C). However, mEHT treatment had no significant effect on either of the other two pro-apoptotic genes (BAX, BAK-1), or the three anti-apoptotic genes tested (Figure 3C). In order to further investigate the contribution of p53 in mEHT-induced cell viability, we combined mEHT with the p53-activating agent nutlin-3a. One day after treatment, mEHT decreased cell viability to 75% (*p* ≤ 0.007), nutlin-3a treatment resulted in 60% viable cells (*p* ≤ 0.0007), while the combined treatment with the two agents induced cell death in 50% of the cells (*p* < 0.0001), indicating that mEHT potentiated the p53-induced cell death (Figure 3D).

**Figure 4:**
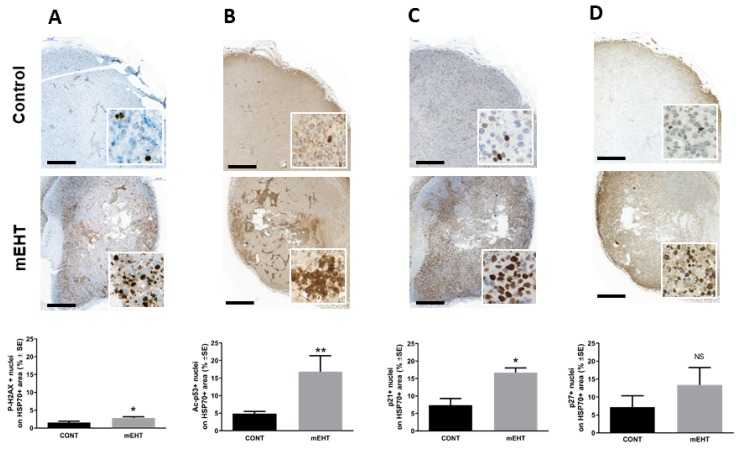
mEHT treatment induced DNA damage, p53 activation, and cell cycle arrest in melanoma tumors. Representative image of tumor sections with immunostaining and below the corresponding quantitative analysis of the percentage of (**A**) p-H2AX, (**B**) Ac-p53, (**C**) p21, and (**D**) p27 on the hsp70+ area detected 24 h after mEHT treatment. *n* = 5 for control and *n* = 6 for mEHT, * *p* < 0.05, ** *p* < 0.004, NS = non-significant, Mann–Whitney test. Scale bar on large images shows 1000 microns, and 45 microns in the insets.

**Figure 5:**
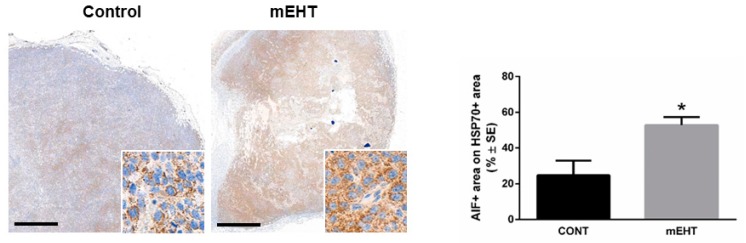
mEHT upregulated the apoptosis inducing factor (AIF). Immunostaining of AIF and corresponding quantitative analysis of the hsp70+ area detected 24 h after mEHT treatment. *n* = 5 for control and *n* = 6 for mEHT, * *p* < 0.05, Mann–Whitney test. Scale bar in large images shows 1000 microns, and 45 microns in the insets.

To investigate the in vivo effect of mEHT on DNA damage signaling and if the induced p53 is involved in melanoma growth arrest, we tested the expression of p53-activation-related proteins using immunohistochemistry. First, we detected γ-H2AX, a marker of DNA double-strand breaks, which can contribute to p53 activation. Modulated EHT treatment resulted in significant upregulation DNA double-strand breaks (*p* < 0.05), as indicated by the upregulation of γ-H2AX in the treated tumors (Figure 4A). In line with this, we demonstrate the mEHT treatment-related stabilization of p53 by the acetylation of p53 (*p* ≤ 0.004) (Figure 4B). Furthermore, our results revealed that mEHT induced DNA damage and p53 activation was accompanied by the upregulation and nuclear localization of proteins responsible for cell cycle arrest. Both the p21^waf1^ (*p* < 0.05) and p27^kip1^ positive tumor cell fractions were highly elevated in response to treatment, although p27^kip1^ elevation did not reach the level of statistical significance (Figure 4C,D). DNA damage, p53 activation, and cell cycle arrest might lead either to cell senescence or programmed cell death. Thus, we characterized the morphological signs and extent of apoptosis, and evaluated the expression of the activated cleaved caspase-3 (cC3) and of the apoptosis-inducing factor (AIF)—effectors of the caspase-dependent and independent pathways. mEHT caused moderate cC3 expression without reaching a significant level (data not shown). On the contrary, significant three-fold cytoplasmic upregulation of AIF levels was detected in the treated tumors (*p* < 0.05) (Figure 5). 

### 2.4. mEHT Induced DAMP Signal Release 

**Figure 6:**
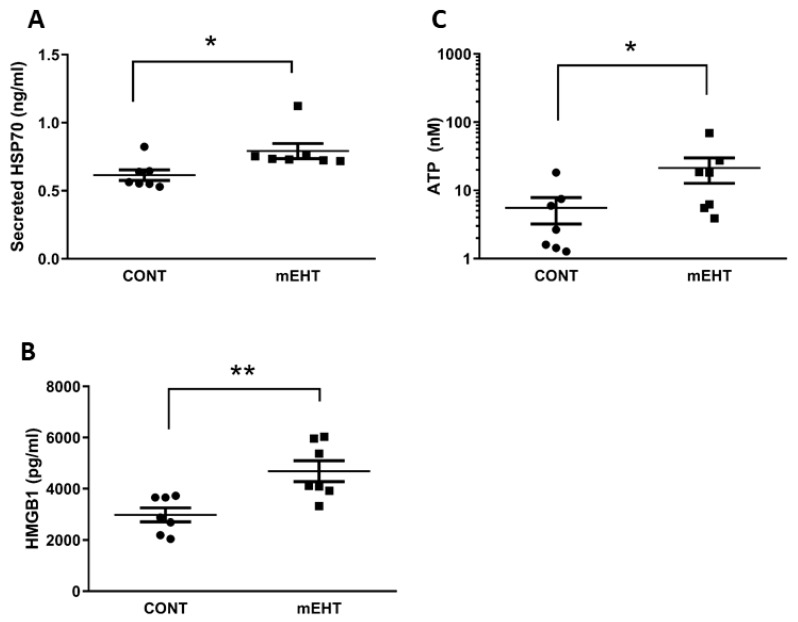
mEHT exposure of the tumors increased the release of damage-associated molecular patterns (DAMPs) into the tumor microenvironment. (**A**) hsp70, (**B**) HMGB1, and (**C**) ATP concentrations were determined in the tumor microenvironment 48 h after the last mEHT treatment using specific ELISA kits for hsp70 and HMGB1, and luciferase-based assay for ATP detection. *n* = 7; * *p* < 0.01 for hsp70, * *p* < 0.04 for ATP, ** *p* < 0.004 were calculated using Mann*–*Whitney test.

The secretion of danger-associated molecular pattern molecules (DAMPs), induced by several anticancer treatments, can mediate immunological cell death (ICD) by promoting anti-tumor immunity. To assess the effect of mEHT on the secretome of the melanoma tumor, we isolated tumor interstitial fluid (see the Methods section) 48 h after the third mEHT treatment, which we used to detect the amount of hsp70, HMGB1 proteins, and ATP. As illustrated in Figure 6, mEHT treatment resulted in a moderate but significant increase in secreted hsp70 (*p* ≤ 0.01), while HMGB1 showed approximately two-fold higher levels (*p* ≤ 0.004) and the concentration of ATP was five-fold higher (*p* ≤ 0.04) in the treated tumors.

### 2.5. mEHT-Related Changes of the Anti-Tumor Immune Response

**Figure 7:**
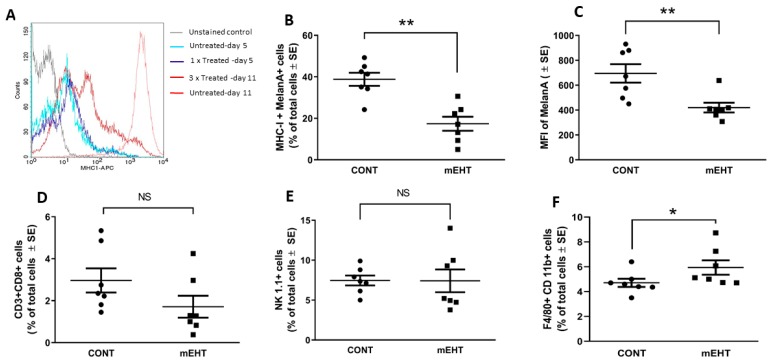
B16F10 tumors downregulated the antigen presentation machinery in response to mEHT treatment, influencing the anti-tumor immune response. (**A**) Comparison of MHC-I expression in melanocytes using flow cytometry after one mEHT treatment on day 5 and after three treatments, on day 11. Tumors were subjected to flow cytometry analysis 48 h after the third mEHT treatment to determine the expression of (**B**) MHC-I and (**C**) melan-A. The percentage of tumor-infiltrating (**D**) cytotoxic T lymphocytes (CD3+CD8+), (**E**) natural killer cells (NK 1.1+), and (**F**) macrophages (F4/80+CD11b+) was determined. ** *p* ≤ 0.004, * *p* ≤ 0.03, NS = non-significant, Mann–Whitney test.

It is well documented that during the course of cytotoxic therapies tumors will activate their escape mechanism by modulating their MHC-I expression [28]. To check if mEHT triggers the immune evasion of melanoma by affecting MHC-I, we conducted flow cytometry analysis on tumor cells after a single and multiple mEHT treatments. Twenty-four hours after a single mEHT treatment, both the treated and control tumor cells expressed low but similar levels of MHC-I (Figure 7A). On day 11 post-inoculation of the tumor cells, 48 h after the third treatment, mEHT induced heterogeneity in the MHC-I expression profile of the tumor, represented by cell populations with low, intermediate, and high MHC-I levels, meanwhile, the non-treated tumor consisted of a single high-MHC-I population (Figure 7A,B). Furthermore, we detected the reduction of melan-A expression and that of MHC-I on treated melanocytes (*p* < 0.004) (Figure 7B,C). Next, we analyzed the immune compartment of the tumors. Consistently with the drop in MHC-I and melan-A expression, we measured a lower percentage of CD8+ T lymphocytes, although the difference was non-significant (Figure 7D). Regarding natural killer cells, both the control and mEHT-treated tumors showed prominent infiltration of NK1.1+ cells, and there was no difference between the two groups (Figure 7E). The percentage of F4/80+CD11b+ macrophages increased significantly in response to treatment (*p* ≤ 0.03) (Figure 7F). 

## 3. Discussion

Modulated EHT, a radiofrequency-induced innovative form of hyperthermia, has been demonstrated in both tumor models and clinical studies to effectively contribute to tumor damage, particularly as a complement to other treatment modalities [2,20,21,26]. The effect of hyperthermia in melanomas has been reported in several studies [29], but the great variability in experimental design makes their comparison with the present work difficult. In this study, we aimed to elucidate the mechanism of action of mEHT treatment on melanoma using both in vitro and in vivo models. First, we set up electric parameters ensuring 42 °C intratumoral temperature throughout the treatment by using invasive temperature monitoring, which indicated a standard temperature gradient, increasing from the skin surface towards the tumor center. This allowed us to maintain the required intratumoral hyperthermia by relying on the temperature measured on the skin surface in later experiments. Then, we demonstrated that the repeated mEHT treatment destroyed tumor cells and inhibited melanoma growth, resulting in significantly reduced tumor weights. This was accomplished by inducing DNA double-strand breaks, which upregulated p53 protein and its downstream cyclin-dependent kinase inhibitors p21^waf1^ and p27^kip1^. The resulting tumor damage led to the release of the DAMP signaling molecules hsp70, HMGB1, and ATP. Although others reported that higher temperatures completely eradicated the primary melanoma tumors [30], it was also shown that although higher temperature (i.e., above 43 °C) was more effective in primary tumor destruction, it completely abrogated the resistance against secondary tumors, most probably by inducing necrotic cell death, which was not effective in mounting anti-tumor immunity [31]. Next, we dissected the molecular pathways leading to tumor regression induced by mEHT. mEHT is known to induce the expression of various heat-shock proteins in different tumor types tested in both in vitro and in vivo models, which could be detected both intracellularly and membrane-localized [20,21,22,24,26,32]. As expected, mEHT activated the heat-shock responses and induced massive hsp70 expression in B16F10 tumors as well, proving the sensitivity and responsiveness of this tumor type to mEHT. To further characterize the cytotoxic effect of the therapy, we looked at its DNA-damaging effects. As B16F10 maintains wild-type p53 (i.e., the master regulator of the stress response and cell death), we first looked at the effect of mEHT on p53 in vitro. We found that hyperthermia resulted in the nuclear translocation of p53. To further describe the downstream events provoked by the p53 activation, we measured the expression of several p53 target genes responsible for cell cycle arrest or apoptosis. The major gene responsible for cell cycle arrest, p21^waf1^, was rapidly and significantly induced by hyperthermia. This is consistent with the data reported by Vancsik [22]. Among the apoptosis-related genes, the most prominent change was observed in the case of PUMA, which was significantly upregulated a few hours after treatment. Our findings are in line with the data published by Vancsik [22], who reported that in the human colorectal cell line C26, mEHT induced the activation and stabilization of p53 by phosphorylation, resulting in swift PUMA mRNA upregulation, cell cycle arrest, and apoptosis [22]. Next, we showed that mEHT had an additive effect on the cell death induced by the p53-activating agent nutlin-3a, demonstrating that mEHT can be a powerful tool for augmenting the tumor-killing effect of treatment modalities targeting p53. After the in vitro findings, we extended our experiments to characterize the DNA damage and p53-related changes in the tumors treated in vivo. It is not clear if conventional hyperthermia has a direct DNA damaging effect or not; it seems more plausible that hyperthermia induces DNA damage by causing protein denaturation and interfering with DNA replication machinery [33]. We measured γ-H2AX to determine the degree of DNA double-strand breaks, and found a significantly higher level in the treated tumors, but whether this elevation was due to a direct DNA-damaging effect of the mEHT remains to be proven.

Although several studies showed that mEHT induces caspase-dependent and independent programmed cell death in various tumor models [20,21,22], in our in vivo model the treatment-induced p53 activation did not lead to apoptosis (neither caspase-dependent nor independent), but it rather induced cell cycle arrest and senescence, marked by the upregulation and nuclear localization of cell cycle inhibitor proteins p21^waf1^ and p27^kip1^, which account for the significant tumor growth inhibition. One of the mediators of caspase-independent cell death is AIF, which in our case, although strongly induced, was sequestered in the cytoplasm, thus hindering cell apoptosis, probably by the intracellular form of hsp70 which is known to form a complex with AIF [34]. Nonetheless, cells expressing high nuclear p21^waf1^ and p27^kip1^ are considered senescent and will be eliminated by phagocytes, the activity of which is known to be enhanced by hyperthermia [35] and the immunogenic environment produced by hyperthermia [21]. We showed that mEHT was effective in producing an immunogenic milieu by triggering the release of ATP, HMGB1, and hsp70, which can promote the anti-tumor immune response [36]. Our data prove that mEHT induces not only intracellular but also membrane-bound and secreted forms of hsp70. While the intracellular hsp70 is known to confer therapeutic resistance [37], the membrane-bound form enhances anti-tumor immunity by helping natural killer (NK) cells to recognize their target cells [38,39]. The extracellular hsp70 was also elevated after mEHT treatment, which facilitates antibody processing and presentation by tagging tumor antibodies for pickup by phagocytes [40]. Tumor cell clearance by cytotoxic lymphocytes and NK cells also depends on the recognition of tumor cells based on their MHC class molecules. Thus, we further evaluated the response of melanoma to mEHT treatment by measuring the expression level of MHC-I. B16F10 melanoma cell line in vitro has low MHC-I expression due to a reversible TAP2 deficiency [41], while in vivo the MHC-I status of this tumor type is regulated by the microenvironment, mainly by IFNγ produced by the tumor-infiltrating NK and γδT cells which are recruited rapidly to B16F10 subcutaneous grafts [42]. Consistent with the previous finding [42], we also show that the surface expression of MHC-I on melanoma altered as the tumor developed, having low expression at an early time point after inoculation (day 5) and increasing progressively during the follow-up of the tumor growth (until day 11). While one single mEHT treatment did not induce changes in MHC-I expression, after the third treatment our results proved that melanocytes responded to the mEHT treatment by reducing their MHC-I molecules. Using flow cytometry, we detected at least three different populations, including high, medium, and low MHC-I-positive clones. Interestingly, similar data were reported when using mechanical stress delivered by a micropump or by ultrasound wave stimuli on the human melanoma cell line. Both caused the detachment and shedding of MHC-I molecules from the cancer cell membranes [43]. It remains to be elucidated if mEHT induces the loss of MHC-I via reversible epigenetic changes in gene transcription, or via post-translational mechanisms. As the low-MHC-I tumor cells became susceptible to NK cytotoxicity, we examined the tumor-infiltrating NK cell population expressing the NK1.1 marker. Although the number of NK cells was not higher in mEHT-treated melanomas, the NK cells can be activated by conventional hyperthermia for enhanced cytotoxicity [44], and “missing MHC-I mediated self-recognition” enables them to kill tumor cells [45,46]. Therefore, in our model, NK cells were likely to contribute to tumor growth control and damage in response to mEHT treatment. However, this needs to be experimentally validated in future studies. Consistent with the loss of MHC-I expression, we also showed that the number of tumor-infiltrating CD8+ cytotoxic T lymphocytes dropped, in parallel with the reduced melanoma MHC-I and melan-A levels. Thus, the observed tumor growth inhibition by mEHT is less cytotoxic T-cell-dependent, although in other tumor models mEHT was found to be effective in stimulating T-cell-dependent tumor immunity [21,24,25]. In line with this, B16F10 melanoma cells were also shown by others to be poorly immunogenic, and CD8+ T-cell-mediated tumor clearance could only be induced in previously immunized hosts [42,47]. However, hyperthermia can support adaptive T-cell-dependent anti-tumor immune response [31]. The pre-immunization of mice under local hyperthermia applied to intradermal B16 melanoma conferred cytotoxic T-cell-mediated host resistance against tumor rechallenge a few days after removal of the primary tumor.

Finally, based on the increased number of F4/80+CD11b+ macrophages, we suggest that this cell population has also an important role in the anti-tumor effect of mEHT. Specifically, the p53-, p21-, and p27-expressing apoptotic/senescent cells in the treated tumors can be cleared away by macrophages [48], whose phagocytic/clearance function is also known to be enhanced by hyperthermia [49].

## 4. Materials and Methods

### 4.1. Cell Culture

The B16F10 mouse melanoma cell line (ATCC^®^ CRL 6475™) purchased from ATCC (Manassas, VA, USA) was cultured in minimum essential medium (MEM) supplemented with 5% (*v/v*) heat-inactivated HyClone fetal bovine serum, 2 mM l-glutamine, 1% (*v/v*) MEM-vitamin solution, 1 mM sodium pyruvate, and 1% (*v/v*) nonessential amino acids (NEAAs) purchased from Thermo Fisher Scientific (Waltham, MA, USA).

### 4.2. Quantitative Real-Time PCR

RNA was isolated from cell using the RNeasy Mini kit (Qiagen, Valencia, CA, USA). RNA concentration and quality were assessed with Nanodrop (Thermo Fischer Scientific). Up to 1 µg of total RNA was converted to cDNA using RevertAid First Standard cDNA synthesis kit (Thermo Scientific). Assessment of mRNA expression was performed by quantitative real-time PCR using cDNA corresponding to 20 ng RNA. PCR reactions were carried out in triplicate, with 300 nM each primer in a final volume of 20 μL of 2× SsoAdvanced™ Universal SYBR Green Supermix (BioRad, Hercules, CA, USA). All primers listed in Table 1 were purchased from Sigma-Aldrich (St Louis, MO, USA). Amplification was performed after one initial denaturation step of 10 min at 95 °C for 40 cycles at 94 °C/10 s and 60 °C/60 s with a CFX Connect™ Real-Time PCR Detection System (BioRad). The fold change of gene expression relative to RPLP0 was defined as 2^−ΔΔCT^.

### 4.3. In Vivo mEHT Treatment

Primary melanoma tumors were established by mixing 1 × 10^6^ B16F10 cells with growth factor reduced matrigel (Trevigen, Gaithersburg, MD, USA) injected subcutaneously into seven-to-nine-week-old female C57Bl/6 mice in the right inguinal area. mEHT treatment was done with a LabEHY 200 device (Oncotherm Ltd., Páty, Hungary). During treatment, mice were laid between plane-parallel asymmetric electric condensers of the circuit. The circuit consisted of a 72-cm^2^ aluminum electrode used as a heating plate set to 37 °C to maintain the physiological body temperature of the animal, and the upper telescopic DIA16 conductive textile electrode, which was placed on the tumor. The impedance was precisely matched, and the electromagnetic field was generated at 13.56 MHz radiofrequency using 1/f amplitude modulation. The first mEHT treatment of 30 min was performed on day 4 after the implantation of the tumor cell/matrigel mixture and was repeated two more times with one day in between. During treatment, mice were anesthetized with 2% isoflurane. Mice were housed with a maximum of four mice per cage, and the treated and control animals were maintained under similar conditions. Animals used in this study were the offspring of C57Bl/6 colonies grown in the animal facility of Semmelweis University, Budapest, Hungary. All animal work conducted during this study was approved by the Governmental Ethical Committee under No.PE/EA/51-2/2019.

### 4.4. In Vitro mEHT Treatment

Cells grown on poly-L-lysine (Sigma-Aldrich) coated coverslips were treated with mEHT for 60 min at 42 °C between two plane-parallel electric condenser plates using a Lab-EHY 100 device (Oncotherm Kft, Budaors, Hungary). After the treatment, coverslip cultures were put into fresh culture medium until further processing. When mEHT was combined with nutlin-3a (Sigma-Aldrich), cells were treated with mEHT for 60 min at 42 °C and then placed in complete medium containing 10 µM nutlin-3a. Twenty-four hours post-treatment, resazurin (Sigma-Aldrich) viability assay was performed. Experiments were repeated at least three times.

### 4.5. Immunohisto- and Cytochemistry

Twenty-four hours after treatment, tumors were fixed in 10% buffered formalin, dehydrated, and embedded in paraffin wax. Three-micron-thick sections were dewaxed and rehydrated prior to hematoxylin–eosin (H–E) staining or immunohistochemistry (IHC). Endogenous peroxidase activity was blocked (15 min in 3% H_2_O_2_ in methanol). For antigen retrieval, sections were heated in Tris-EDTA (TE) buffer pH 9.0 (0.1 M Tris_base and 0.01 M EDTA) in a pressure cooker (2100 Antigen Retrieval, Aptum Biologics Ltd., Southampton, United Kingdom) for 20 min, followed by a 20 min cooling. After blocking the non-specific protein binding sites in 0.1 M Tris-buffered saline (TBS, pH7.4) containing 3% bovine serum albumin (BSA, #82-100-6, Millipore, Kankakee, Il, USA), 0.1% Tween-20 (Sigma-Aldrich, St Luis, MO, USA) and 0.01% sodium azide for 20 min, slides were incubated in a humidity chamber overnight (16 h) using the antibodies listed in Table 2, diluted in 1% BSA/TBST. For p53 (acetyl K386) the pH 6.1 Target Retrieval Solution (#S1699, TRS, Dako, Glostrup, Denmark) was used for antigen retrieval and 5% normal goat serum (#S26-100ML, Merck, Darmstadt, Germany) for protein block. A Histols^®^ micropolymer-peroxidase-conjugated anti-rabbit Ig detection system (#30011.500T, Histopathology Ltd., Pécs, Hungary) was applied for 40 min and the reaction developed using the DAB Quanto Chromogen and Substrate kit (#TA-060-QHDX, Thermo, WA, USA). All incubations were at room temperature, and the samples were washed between incubations for 3 × 3 min in TBST (pH 7.4). Slides were digitalized and the immuno-reactions were evaluated using the modules of the QuantCenter image analysis software tool pack (3DHISTECH, Budapest, Hungary). Hsp70 positivity indicated areas of early cell stress, which were focused on for identification of the molecular changes in the mEHT-treated tumors. Since the control samples did not show relevant hsp70 upregulation, the whole tumor area was annotated for calculations. Positive areas for cleaved caspase-3 and hsp70 expression were determined as the percentage of the whole annotated areas (HistoQuant module). The portion of AIF, phospho(Ser139)-H2AX-, p21^waf1^-, and p27^Kip1^-positive cell nuclei were calculated from the gross number of tumor cell nuclei (CellQuant module).

Vendor specification: Cell Signaling (Danvers, MA, USA), Thermo Fischer Scientific, Dako (Glostrup, Denmark), Sigma-Aldrich, Abcam (Cambridge, United Kingdom).

For immunocytochemistry coverslip cultures were fixed in 4% paraformaldehyde/PBS solution for 10 min at 4 °C then flushed three times with PBS. Samples were permeabilized with TBS containing 0.3% Tween-20 (#P9416, Sigma-Aldrich; 0.3% TBST) for 20 min, then washed with 0.1% TBST. Blocking procedure was followed by incubation with goat polyclonal antibody for p53 (1:350, #AF1355, Bio-Techne, Minneapolis, MN, USA). For immunofluorescence Alexa Fluor 546 (orange-red) coupled anti-goat IgG (1:200, #A11056) was used for 60 min, then cell nuclei were stained blue with 4′,6-diamidino-2-phenylindole (DAPI) (both from Thermo Fischer Scientific).

### 4.6. Flow Cytometry

Tumors were digested with Liberase (Roche, Basel, Switzerland) supplemented with DNase (Sigma-Aldrich, St Louis, MO) solution for 45 min at 37 °C and filtered with a 70-μm cell strainer (Corning, New York, USA) to obtain single-cell suspension. Cells were centrifuged and treated with red blood cell lysis buffer (Biolegend, San Diego, USA) for five minutes at room temperature to remove the erythrocytes. Cells were washed and re-suspended in staining buffer (PBS supplemented with 0.1% BSA). FcR blockade was performed by incubation with TruStain fcX (Biolegend). After cell count, 1 × 10^6^ cells were stained for 30 min at 4 °C with the following antibodies: Melan-A (Abcam) conjugated with PE and MHC class I conjugated with APC (eBioscience) was used to determine the percentage of MHC-I-expressing melanoma cells in the tumor. Intracellular hsp70 was determined with hsp70-FITC antibody (Abcam). Intracellular staining for hsp70 and melan-A antibodies was performed with the intracellular fixation and permeation buffer set (eBioscience) following the protocol advised by the kit. Membrane-expressed hsp70 was determined with cmhsp70.1-FITC (Multimmune GmbH, München, Germany). Tumor-infiltrating cytotoxic T lymphocytes were detected using CD3-PE and CD8-APC for cytotoxic T lymphocytes (Biolegend). Natural killer cells were measured using NK1.1-PE, macrophages by using F4/80-FITC, CD11b-PE (Biolegend). Samples were measured with a flow cytometer (FACSCalibur, BD Biosciences), and the data were analyzed with CellQuest Pro Software (BD Biosciences).

### 4.7. Measurement of Extracellular hsp70, HMGB1 and ATP

The tumor interstitial fluid was obtained by digesting tumors as described above. After removing cells by centrifugation, the supernatant was further purified by centrifugation at maximum speed for 10 min and stored at −70 °C until use. The protein concentration of the tumor effusate was measured using Bradford reagent (Thermo Fisher Scientific). In the subsequent measurements, an equal amount of protein was used. The hsp70 concentration was measured using a hsp70 High-Sensitivity ELISA kit (Abcam). The HMGB1 concentration was determined using a specific ELISA kit from Aviva Systems Biology (San Diego, CA, USA) in accordance with the manufacturer’s protocol. Absorbance was measured at 450 nm with PowerWave microplate spectrophotometer (BioTek, Winooski, VT). ATP content was evaluated using a luciferase-based ATP determination kit (Thermo Fischer Scientific) following the manufacturer’s instructions. Luminescence was measured with a Varioskan Flash microplate reader (Thermo Fischer Scientific).

### 4.8. Statistical Analysis

Statistical analysis was performed using GraphPad Prism software (v.6.07; GraphPad Software Inc., La Jolla, CA, USA). Unpaired Student’s *t*-test was performed for statistical comparison between treated and control tumor weights. In experiments with more than two groups, statistical differences were compared with one-way ANOVA. Mann–Whitney nonparametric test was performed for all other comparisons. Data are expressed as mean ± SE. *p*-values < 0.05 were considered significant.

## 5. Conclusions

In this study, we demonstrated that mEHT treatment (at 42 °C for 30 min) could inhibit melanoma growth despite downregulating MHC-I levels and tumor antigen presentation. mEHT treatment could induce hsp70 protein overexpression in melanomas involving their cell membranes, which activated p53 and cyclin-dependent kinase inhibitors p21^waf1^ and p27^kip1^ to promote cell cycle arrest and tumor cell senescence. Hsp70 release could also support tumor destruction through activating NK-cells, while senescent tumor cells were cleared away by elevated numbers of macrophages. Although our earlier and recent results showed that mEHT can induce tumor damage alone, combining it with already existing foundational treatment modalities is likely to strengthen their efficiency against cancer, including melanoma. Clarifying the mechanism of action of mEHT treatment in different tumor types can support the design of more efficient treatment modalities combining mEHT with conventional targeted molecular therapy or immunotherapy.

## Figures and Tables

**Table 1:** Primer sequences used in this study.

Gene	Forward Primer (5′–3′)	Reverse Primer (5′–3′)
***RPLP0***	CTCTCGCTTTCTGGAGGGTG	ACGCGCTTGTACCCATTGAT
***BCL-2***	CTCGTCGCTACCGTCGTGACTTCG	CAGATGCCGGTTCAGGTACTCAGTC
***BCL-XL***	AACATCCCAGCTTCACATAACCCC	GCGACCCCAGTTTACTCCATCC
***BAX***	AAGCTGAGCGAGTGTCTCCGGCG	GCCACAAAGATGGTCACTGTCTGCC
***BAK-1***	CAGCTTGCTCTCATCGGAGAT	GGTGAAGAGTTCGTAGGCATTC
***XIAP***	ATGCTTTAGGTGAAGGCGAT	CATGCTGTTCCCAAGGGTCT
***PUMA***	TCTATGGGTGGAGCCTCAGT	GAGGGCTGAGGACCCATTAAA
***p21***	GCAGAATAAAAGGTGCCACAGG	AAAGTTCCACCGTTCTCGGG

**Table 2:** Antibodies and conditions used for immunohisto- and immunocytochemistry.

Antigen	Type	Reference	Dilution	Vendor
AIF	Rabbit, pAb	#4642	1:70	Cell Signaling
p-H2AX(Ser139)	Rabbit, mAb	#9718	1: 200	Cell Signaling
Cleaved caspase-3	Rabbit, pAb	#9664	1:300	Cell Signaling
Hsp70	Rabbit, pAb	#4872	1:200	Cell Signaling
P21^waf1^	Rabbit, mAb	#ab188224	1:500	Abcam
P27^kip1^	Rabbit, pAb	# RB9019P	1:50	Thermo
P53 (acetyl K386)	Rabbit, pAb	#ab52172	1:200	Abcam
P53	Goat, pAb	#AF1355	1:350	Bio-Techne
